# A Highly Sensitive Urinary Exosomal miRNAs Biosensor Applied to Evaluation of Prostate Cancer Progression

**DOI:** 10.3390/bioengineering9120803

**Published:** 2022-12-14

**Authors:** Yueh-Er Chiou, Kai-Jie Yu, Sow-Neng Pang, Yan-Lin Yang, See-Tong Pang, Wen-Hui Weng

**Affiliations:** 1 Department of Nursing, College of Medicine, Fu Jen Catholic University, New Taipei City 242, Taiwan; 2 Department of Chemical Engineering and Biotechnology and Graduate Institute of Biochemical and Biomedical Engineering, National Taipei University of Technology, Taipei City 106, Taiwan; 3 Division of Urology, Department of Surgery, Chang Gung Memorial Hospital, Taoyuan 33305, Taiwan; 4 Department of Emergency Medicine, Monash Medical Centre, Melbourne, VIC 3083, Australia

**Keywords:** prostate cancer, SPCE, microRNA, biosensor

## Abstract

Prostate cancer is the most common cancer in the male population, carrying a significant disease burden. PSA is a widely available screening tools for this disease. Current screen-printed carbon electrode (SPCE)-based biosensors use a two-pronged probe approach to capture urinary miRNA. We were able to successfully detect specific exosomal miRNAs (exomiRs) in the urine of patients with prostate cancer, including exomiR-451 and exomiR-21, and used electrochemistry for measurement and analysis. Our results significantly reaffirmed the presence of exomiR-451 in urine and that a CV value higher than 220 nA is capable of identifying the presence of disease (*p*-value = 0.005). Similar results were further proven by a PAS greater than 4 (*p*-value = 0.001). Moreover, a higher urinary exomiR-21 was observed in the high-T3b stage; this significantly decreased following tumor removal (*p*-values were 0.016 and 0.907, respectively). According to analysis of the correlation with tumor metastasis, a higher exomiR-21 was associated with lymphatic metastasis (p-value 0.042), and higher exomiR-461 expression was correlated with tumor stage (*p*-value 0.031), demonstrating that the present exomiR biosensor can usefully predict tumor progression. In conclusion, this biosensor represents an easy-to-use, non-invasive screening tool that is both sensitive and specific. We strongly believe that this can be used in conjunction with PSA for the screening of prostate cancer.

## 1. Introduction

Prostate cancer (PCa) has become the most formidable disease and cancer for men. As the most common cancer in men, there were 1.4 million incident cases in 2016, with more than 380,000 deaths. With the aging population, incidents also increased significantly by more than 40% over a 10-year period from 2006 to 2016. The odds of developing prostate cancer was 1 in 16 globally [1]. This disease becomes even more threatening for men over 50 years old, with 99.9% of cases occurring after this age threshold [2]. Therefore, early identification of disease is essential to help patients receive prompt treatment, which could significantly improve morbidity and mortality rates [3,4]. In the current climate of screening for PCa, we are limited to accurate and consistent histories from patients and clinicians’ physical examination findings. Additionally, clinicians employ a serum marker called prostate-specific antigen (PSA) as a main indicator for the presence of prostate disease [5]. This is followed by biopsies and the use of the Gleason score (GS) to determine prognosis [6]. Although biopsies are mostly definitive, they can be incredibly invasive and therefore not suitable as a universal screening procedure. PSA, although crucial in PCa screening, has been heavily criticized. A systematic review of the diagnostic accuracy of PSA screening suggested an unsurprisingly high overdiagnosis rate, with 1.5 to 2-fold false-negative rates [7,8]. This can be attributed to a multitude of diseases and activities that can cause fluctuations in PSA levels, including medications; exercise; prostate stimulation, including sexual activity and ejaculation; urinary tract infection; prostatitis, etc. [9,10,11,12,13,14]. As such, enhancing accuracy in the screening for prostate cancer has become an increasingly popular subject.

We have seen an exponential growth in the use of biosensors in all fields of study, including cancer research [15], environmental pollution monitoring [16], and, most recently, in the detection of SARS-CoV-2 [17]. In the field of cancer screening and detection, designs most commonly utilize complementary nucleic acid sequences, antibodies for detection, or other molecules annealed to the surface of the transducer. Different techniques are used, including electrochemical detection, optical detection, and mass-based detection techniques for data interpretation [18]. Relative to older detection techniques, including reverse-transcriptase quantitative polymerase chain reaction (RT-qPCR), Northern blotting, in situ hybridizations, and deep sequencing [19], they differ significantly from a user’s point of view. Older technology requires more extensive preprocessing of samples with convoluted protocols, making it unsuitable for point-of-care use or immediate analysis. This is unlike modern biosensors, which are more portable, convenient, and cost-effective as screening tools.

In the current climate of genetics research, microRNAs have become an interesting focus. They are small, non-coding RNAs that inhibit post-translational gene expression by targeting messenger RNAs (mRNA) [20], with direct implications for regulation of cell proliferation, differentiation, and apoptosis. miRNAs are particularly interesting in cancer research due to potential roles in tumor suppressor genes and oncogenes [21], depending on the expressional type of individual miRNA [22,23]. The properties of miRNA also make it suitable for screening, as it is widely present in all tissues and bodily fluids, including serum, plasma, and urine [24]. Furthermore, following secretion into circulation through exosome microvesicles, the robust nature of the encapsulated miRNA, as well as the length of the nucleic acid, enhances its resistance to degradation through endogenous RNase [23,25]. As such, its intuitive applications prompted this experiment. Several studies have demonstrated the importance of miRNAs and their links to disease and cancers [17,26,27].

The association of one miRNA with disease is simply not precision medicine, so to tackle this problem, multiple-miRNA profiling is used to provide more information about cancer [28,29]. In this study, urinary exomiRNA-21 and exomiRNA-451 were chosen, owing to their association with PCa. A previous study showed that the expression of both urinary exomiR-21 and exomiR-451 was significantly increased in patients with metastatic PCa [30]. Given the properties of miRNA, we study chose to use urine samples rather than serum samples, owing to its ease of access, ease of collection, and non-invasiveness, which can translate into the clinical setting. The principle of this platform design is based on a screen-printed carbon electrode with optimized surface modification, as well as the use of a two-pronged probe made of complementary single-stranded DNA (ssDNA) to capture target exomiR-21 or exomiR-451. The signal is transduced through electrochemistry. Through signal amplification with enzymes, the current response is quantified using chronoamperometry (CA), differential pulse voltammetry (DPV), cyclic voltammetry (CV), and electrochemical impedance spectroscopy (EIS) [31].

## 2. Materials and Methods

### 2.1. Urine Samples Collection

In total, forty-three urinary samples were collected from twenty-two adenocarcinoma PCa patients (of these, pre- and postoperative urine samples were collected in ten cases) and two benign prostatic hyperplasia (BPH) patients, and ten control samples were collected from young, healthy men in this study. The timing of urine collection was before the operation and day 7 post operation, with no specific instructions for collection. Patient recruitment and sample collection were performed appropriately as per human subjects protocol approval from the Institutional Review Board at Cheng Gung Memorial Hospital, Taoyuan, Taiwan, with statements of informed consent (IRB No: CMRPG3K0621).

### 2.2. Reagents and Chemicals

**Figure 1:**
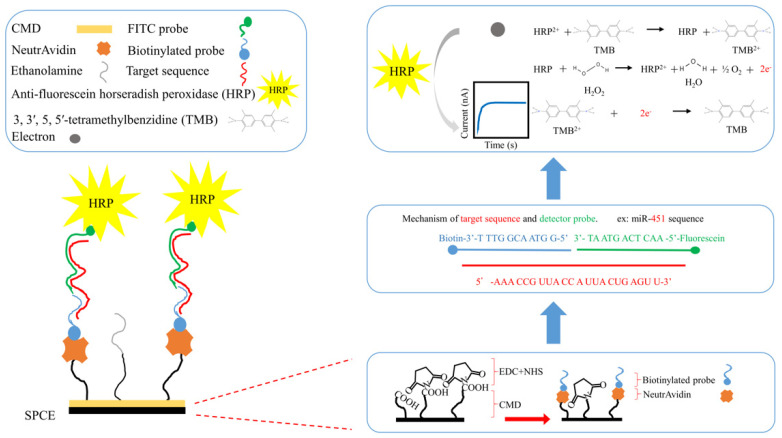
Schematic diagram of the sensor manufacturing process. NeutrAvidin and biotinylated ssDNA probe-modified, screen-printed carbon electrode (SPCE)-based sensor strip and its use for the detection of exosomal microRNAs.

The experimental material consisted of a screen-printed carbon electrode (SPCE) as a sensing platform, which was purchased from Zensor R&D (Taichung, Taiwan). Figure 1 shows our previous developed biosensor with optimizations to enhance signal detection [17]. The following materials and reagents were purchased from Sigma-Aldrich (Sigma-Aldrich St. Louis, MO, USA): phosphate-buffered saline (PBS), carboxymethyl dextran sodium salt (CMD-Na), 1-ethyl-3-(3-dimethylaminopropyl) carbodiimide (EDC), N-hydroxysulfosuccinimide (NHS), ethanolamine, potassium chloride (KCl), potassium ferricyanide (Fe(CN)_6_^3−^), hydrogen peroxide (H_2_O_2_), 3, 3′, 5, 5′-tetramethylbenzidine (TMB), and diethyl pyrocarbonate (DEPC). NeutrAvidin protein was supplied by Thermo Fisher Scientific (Waltham, MA, USA). Antifluorescein horseradish peroxidase (HRP), a 40,000 dalton protein, was acquired from Abcam (Cambridge, UK). MES-free acid monohydrate, hydroxymethyl-aminomethane (Tris), and ethylenediaminetetraacetic acid (EDTA) were purchased from Amresco (Amresco Inc., Solon, OH, USA). Sodium chloride (NaCl) was obtained from Promega Corporation (Madison, WI, USA). We designed a biotinylated ssDNA probe (bioreceptor probe), fluorescein (FITC) ssDNA probe (detector probe), and artificial mimic targeting sequence using Genomics (Taipei, Taiwan).

### 2.3. Methods and Apparatus

#### 2.3.1. Characterization of SPCE by SEM

Stepwise electrode surface modification was verified at each stage. Scanning electron microscopy (SEM; HITACHI S-3000H, Japan) was used first to determine the modification effects of each step; then, using cyclic voltammetry (CV), the stability, sensitivity, and selectivity of the modified SPCE were confirmed. Finally, the urine samples of exosomal miRNAs were evaluated by chronoamperometry (CA).

#### 2.3.2. Electrochemical Analysis

All electrochemical experiments were carried out using the AutoLab PGSTAT204 electrochemical workstation (AutoLab PGSTAT204, Metrohm, Herisau, Switzerland). The CV used a standard three-electrode electrochemical system consisting of a modified SPCE as a working electrode and a silver/silver chloride (Ag/AgCl) and a platinum wire as the reference electrode and counter electrode, respectively. Based on our previous studies, similar electrochemical parameters were used as references, with minor modifications [32]. In addition, two electrochemical methods, CV and CA, were performed for sample detection. Under CV, the test was carried out in 5 mM ferricyanide and 0.1 M KCl solutions. A ferricyanide solution containing KCl was utilized as a mediator to generate electron flow from redox reactions between potassium ferricyanide (K3[Fe(CN)6]) and potassium ferrocyanide (K4[Fe(CN)6]). The CV potential setting in SPCE ranged from −0.3 to 0.8 V at a scan rate of 50 mV/s. Then, 0.4 mM TMB and 0.4 mM H_2_O_2_ solution was applied for CA measurement with a setting of −200 mV against a saturated calomel electrode; the scan rate was 50 mV/s, and the electroreduction current was measured 150 s after the HRP redox reaction reached a steady state.

#### 2.3.3. Biotinylated Capture Probe and FITC Detector Probe Design

The principle behind this exomiRNA biosensor is based on two homemade short nucleotide sequences for respective miRNAs, labeled as a capture probe and a detector probe, as illustrated in Figure 1. Capture probes are modified with biotin at the 3′ end of the sequence and affixed to the surface of the SPCE. Detector probes are modified with fluorescein (FITC) at the 5′ end of the sequence and designed to bind to horseradish peroxidase (HRP), for example, miR-451 capture probe: 5′-GGT AAC GGT TT-3′-biotin and corresponding detector probe: FITC-5′-AACTCAGTAAT-3′; or miR-21 capture probe: 5′-CTGATAAGCTA-3′-biotin and corresponding detector probe: FITC-5′-TCA ACA TCA GT-3′. Both probes aim to anneal with the corresponding sequences on the target miRNA; once a stable double-stranded structure has been formed, the signal generation process would be carried out by HRP and substrate 3,3′,5,5′-Tetramethylbenzidine (TMB) via a redox reaction; and through the transfer of electrons, signal was recorded through CV.

#### 2.3.4. Modification of SPCE

The SPCE surface was designed according to our previous study on urinary miRNA sensing in colorectal cancer, with further optimizations [27]. In brief, we used 50 μL of CMD-Na (50 mg/mL) to generate a carboxylic (COOH) functional group on the SPCE surface and dipped it into a solution of 8 mg/mL 1-ethyl-3-(3-dimethylaminopropyl) carbodiimide (EDC) and 22 mg/mL N-hydroxysulfosuccinimide (NHS) in a 0.1 M MES buffer (pH 4.7) for 15 min at room temperature in order to activate the COOH, resulting in a stable RC=O-NH-R_2_ bond, which allows NeutrAvidin to bind covalently. Finally, a 5 μM biotinylated single-stranded oligonucleotide sequence (ssDNA) or a capture probe containing a partially mimicked complimentary exomiR-451 or exomiR-21 sequence was conjugated. The completed functional surface of a “biotinylated receptor” was formed, which is complementary to the corresponding terminal sequence of the targeted urinary exosome microRNA. Then, 1M of ethanolamine was applied to occupy the inactivated sites of the SPCE surface, and 0.1% DEPC-treated water was prepared to inactivate RNase and ensure the stability of the reaction. This completed the modification of the SPCE, making it functionalized and ready-to-use.

### 2.4. Isolation and Quantification of MicroRNAs from Urinary Exosomes

The total RNA and all sizes of exosomal RNAs (exomiR) from urine were extracted using Norgen’s Urine MicroRNA Purification Kit (#29000, Norgen Biotek, Thorold, ON, Canada). The process performed was in accordance with the manufacturer’s protocol, with minor modifications; only small RNAs with a molecular size of less than 200 nt were isolated and purified. In brief, 1 mL of urine per sample was lysed and centrifuged at 8000× *g* rpm. Samples were washed twice and centrifuged at 14,000× *g* rpm. The purified miRNA was eluted using 50 μL elution buffer and stored at −80 °C until used. The final concentration yield was dependent on the specimen; the mean RNA concentrations were around 30 ng/10 μL.

### 2.5. Statistical Evaluation

Charts were generated with Excel 2016 and Origin 9.0, and statistical analysis was performed using GraphPad Prism version 8 and IBM SPSS Statistics 23 to compare test and control samples and calculate *p*-values, with values less than 0.05 considered statistically significant.

## 3. Results

### 3.1. Characterization of exomiR-SPCE-Based Biosensor

#### 3.1.1. Characterization of Modified SPCE by Scanning Electron Microscopy (SEM)

**Figure 2:**
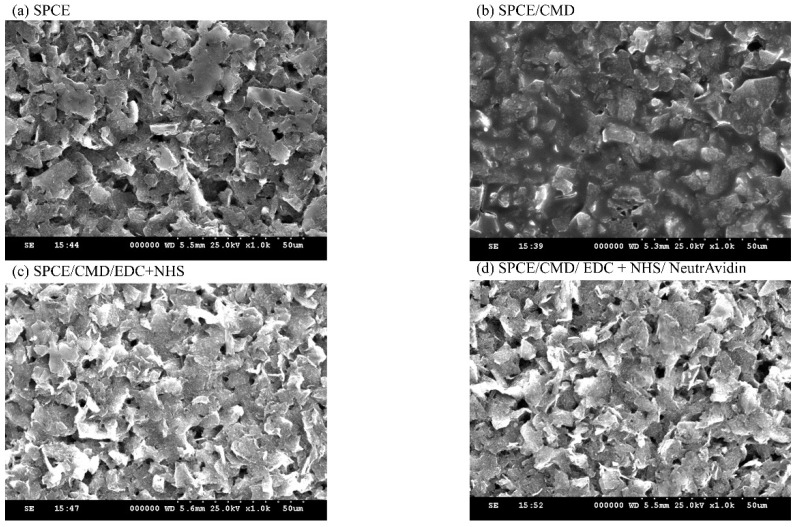
Scanning electron microscopy (SEM) images of stepwise modification of a screen-printed electrode (SPCE). (**a**) Bare SPCE without modification. (**b**) After CMD modification. (**c**) After modification with EDC and NHS. (**d**) Modification with NeutrAvidin.

SEM was performed to examine the morphologies of the modified SPCE surface; the results are shown in Figure 2. After treatment with CMD-Na (50 mg/mL), carboxylic (COOH) functional groups were generated. The surface was then dipped in EDC and NHS to activate the COOH, resulting in a stable RC=O-NH-R_2_ bond that allowed for the subsequent addition of NeutrAvidin to bind covalently. Due to the mutual crosslinking of CMD molecules, the rough edges seen in Figure 2a change to a smooth and vague edge border in Figure 2b. After treatment with EDC/NHS, the edges became curled, indicating activation by EDC/ NHS and the effect of crosslinking (Figure 2c). The final modification with NeutrAvidin seen in Figure 2d demonstrates a smoother and clean surface.

#### 3.1.2. Characterization of ExomiR-SPCE by Cyclic Voltammetry

**Figure 3:**
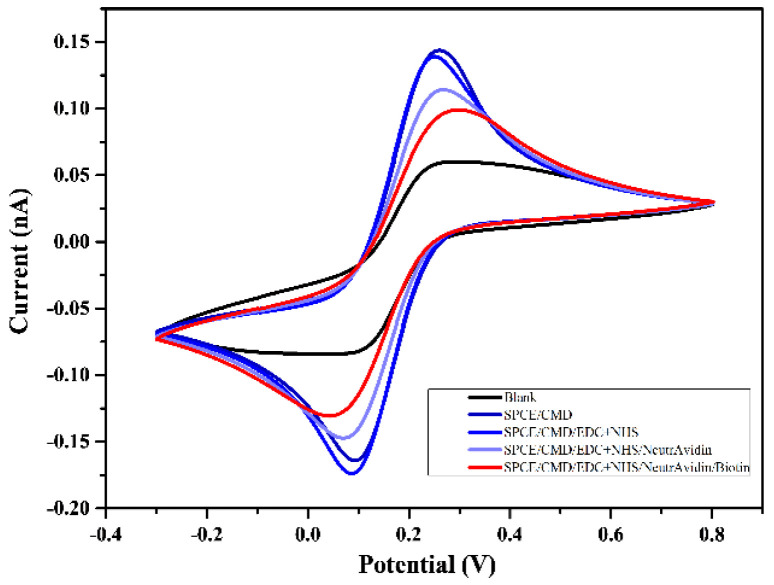
Cyclic voltammetry of SPCE surface modification at every step, with a scan rate of 0.05 V/s and a range of −0.3~0.8 V.

In addition, to ensure every step of SPCE surface modification was successfully completed, various grading current signals were measured by cyclic voltammetry (CV). The surface of the SPCE was first coated with electrodes in various chemical functional groups; then, the CV operation was performed to observe the changes after modifications. A scan rate of 0.05 V/s and range a of −0.3~0.8 V were applied for scanning analysis and verification in the following steps: unmodified SPCE (Figure 3; black line indicates blank), post activation by CMD (denoted as SPCE/CMD), post NHS + EDC solution reaction (denoted as SPCE/CMD/EDC + NHS), NeutrAvidin solution reaction (denoted as SPCE/CMD/EDC + NHS/NeutrAvidin), and biotin probe reaction (denoted as SPCE/CMD/EDC + NHS/NeutrAvidin/Biotin) (Figure 3). Each step showed a clear difference in current, confirming that every treatment successfully modified the surface of the screen-printed electrodes. The final modifications of reduction peaks and oxidation peaks occurred at 0.1 V and 0.3 V, respectively (Figure 3, red line).

### 3.2. Characterization of Biosensor Sensitivity

**Figure 4:**
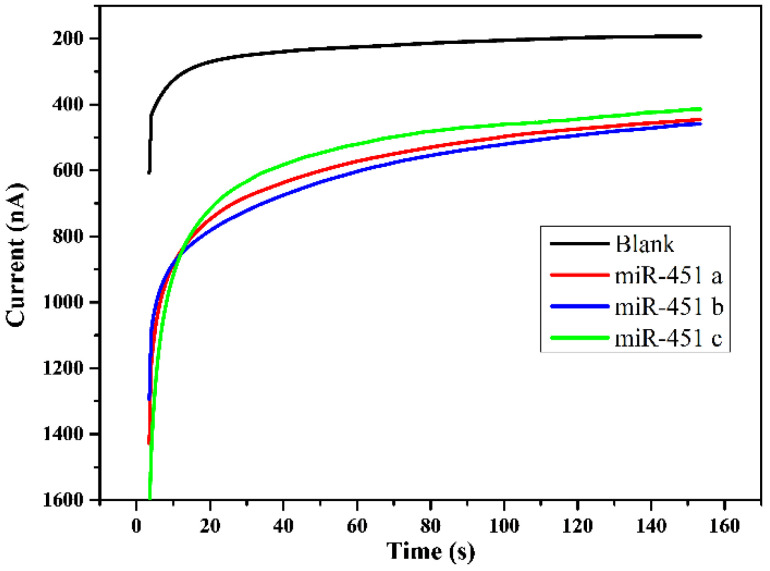
The sensitivity of the exomiR-biosensor was confirmed by urine samples. A total of 10 nM of exomiR-451 was derived from the urine of three patients for triple testing. The resulting CA value shows clear cutoffs relative to the control test.

Sensitivity of the exomiR-biosensor was confirmed by clinical urine samples. An average of 10 nm of exomiR-451 was derived from the urine of three patients. After triple testing (Figure 4), all CA curves remained in a consistent range of results compared to the blank control test, representing a clear difference. First, the target exomiR-451 bound to the FITC probe, then further caught by the biotin probe that had previously been fixed on the SPCE after 150 s of sequences interactions at a voltage of 0.3 V caused the reaction to activate the horseradish peroxide and hydrogen peroxide, further releasing the electrolyte to be detected (Figure 1 and Figure 4). The results indicate that after successfully capturing the target exomiR, the current usually stably reaches up to 500 nA (Figure 4).

### 3.3. Characterization of Sensor Selectivity

**Figure 5:**
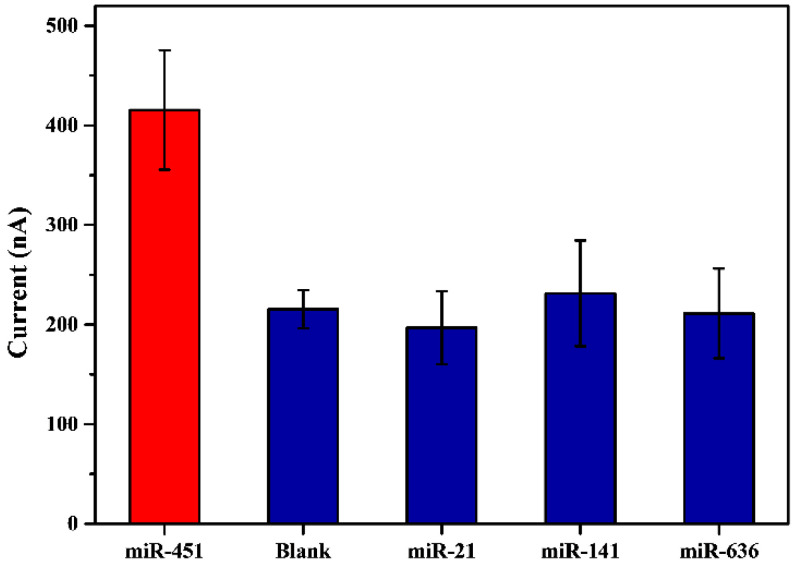
Test for selectivity of the miR-451 biosensor against random exomiRs, including miR-21, miR-141, and miR-636, as well as a negative control.

To ensure adequate selectivity of the biosensor for targeted miRNA, the designed exomiR-451 biosensor was tested against random miRNAs, including miR-21, miR-141, and miR-636, as well as a blank for control. The results clearly demonstrate the effectiveness of target exomiRNA capture. All non-targeted exomiRNAs failed to produce a strong current, with currents similar to the blank test of approximately 200 nA. Conversely, the test with the targeted exomiR-451 produced a current signal at 430 nA with no overlapping standard deviations with its comparison (Figure 5).

### 3.4. Test for Concentration of Urinary ExomiRNAs by exomiR-biosensor

**Figure 6:**
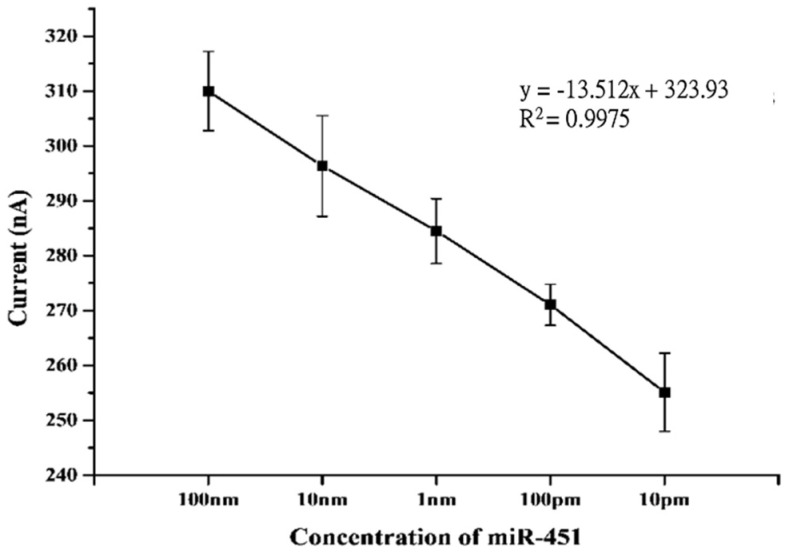
Illustration of the electrochemical generation of a concentration gradient of miR-451. Variation of the concentration profiles as a function of current and the concentrations from 10 pM to 100 nM were tested. The results show that the concentration gradient is linear, and R^2^ = 0.9975.

The expression levels of miRNAs are highly correlated with certain gene expressions that might reflect disease status. In order to accurately measure the total amount of exomiR in urine, the exomiR biosensor was tested by gradient concentrations of 100 nM, 10 nM, 1 nm, 100 pM, and 10 pM, producing a linear regression with an R value of 0.9975. The results demonstrated biosensor consistency and stability throughout a range of concentrations of miRNAs (Figure 6).

### 3.5. Comparison of Preoperative and Postoperative (7 Days) Levels of exomiR-21 and exomiR-451

**Figure 7:**
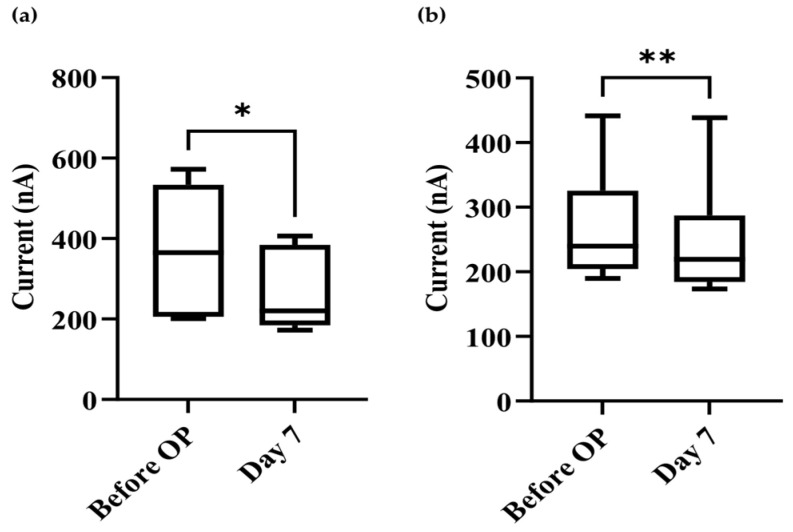
Comparison of preoperative (Before OP) and 7-day postoperative (Day 7) currents by T-test analysis using 10 nM sample concentration. (**a**) miR-21, * represented *p*-value = 0.0203; (**b**) miR-451, ** represented *p*-value = 0.0081.

We compared the preoperative and 7-day postoperative phases of exomiR-21 and exomiR-451. The results indicate that 7 days post tumor resection, both exomiR-21 and exomiR-451 had significantly decreased, with *p*-values of 0.0203 and 0.0081, respectively (Figure 7).

### 3.6. Statistical Analysis of Correlation between Clinical Characteristics and exomiR-21 and exomiR-451 Expression in Urine

We further analyzed the correlation between the expression levels of urinary exomiR-21 and exomiR-451 with the clinical characteristics of preoperative patients, including disease type, age of the patient, tumor stage, TNM stage, and Gleason score. By setting the current to 220 nA as a threshold and employing the biomarker exomiR-451, we were able to distinguish prostate disease type between adenocarcinoma, BPH, and normal patients. As seen in Table 1, all non-cancerous subgroups were under the threshold of 220 nA, and the majority of adenocarcinoma cases was significantly above the 220 nA threshold, with a *p*-value of 0.005. Similar results were observed when categorizing established PSA parameters using current thresholds at 220 nA. All categories of patients with a PSA ≤ 4 ng/mL fell under the 220 nA threshold, and the majority of patients with a PSA ≥ 4 ng/mL was above the 220 nA threshold (*p*-value = 0.001). Interestingly, after grouping patients into PSA levels of above or below 10 ng/mL, there is still significant evidence that a higher PSA level is correlated with higher current values and vice versa (*p*-value = 0.037), whereas applying the same parameters to miRmiR-21 biomarker to determine prostate cancer staging and age, results were not significant (Table 1).

We further applied this biosensor to detect exomiR-21 and exomiR-451 in the urine of ten patients before and after operation. Based on the difference between the preoperative and postoperative periods and statistical analysis of clinic pathological data, it can be observed that the category of tumor size with preoperative exomiR-21 had statistical significance for pT2, pT3a, and pT3b, with a *p*-value of 0.016. In the presence or absence of lymphatic metastasis, the preoperative and postoperative difference of exomiR-21, the *p*-value was 0.042. With respect to tumor stage, the preoperative and postoperative difference of exomiR-451, the *p*-value was 0.031. Application of the biosensor in these three aspects has considerable statistical significance (Table 2).

## 4. Discussion

Following the trend in the field of cancer research and biosensors, we proposed an interesting combined miRNA biosensor to detect exomiR-21 and exomiR-451 in association with PCa, utilizing a well-established platform that has proven effective in the detection of multiple miRNAs [32]. The construct is simple and non-invasive screening tool that is operable with minimal training. The results presented in this study demonstrate a stable platform for target miRNA detection. The main purpose of the exomiR biosensor is to provide another simple screening tool and diagnostic marker for PCa other than PSA.

The significant results obtained using the miR-451 exomiR biosensor are strongly suggestive of a reliable new screening method for PCa in conjunction with PSA, although exomiR-21 was unable to provide significant evidence for disease differentiation (Table 1). Current values at or above the 220 nA threshold differentiate between samples with a low PSA concentration and those with a normal prostate or with BPH, enabling further investigation of current values above the 220 nA threshold, which can improve the detection rate of PCa, as PSA is an unreliable screening test for PCa. In addition, we further attempted to establish the significance of exomiR-21 and exomiR-451 in postoperative care. Our results showed that the levels of exomiR-21 and exomiR-451 were significantly different between the preoperative phase and the 7-day postoperative phase.

An appropriate scan rate of 0.05 V/s and a range of −0.3~0.8 V were applied to the exomiR biosensor for scanning analysis and verification (Figure 3). The detected signals were derived from electrolytes that were released from the target miRNA sequence bound to the FITC probe and biotin probe, which required approximately 150 s at a voltage of 0.3 V (Figure 1 and Figure 4) and a stable current reaching 500 nA (Figure 4). The sensing reproducibility was also verified by testing urine samples from three different patients with 10 nM of exomiR-451; all curves were consistent with a current of approximately 500 nA, compared with the resulting CA value, which showed clear cutoffs relative to the control test (Figure 4). Furthermore, the high selectivity of the targeting probe design was proven by a mixture containing at least four different sequences, with the highest current value only occurring with the targeting miRNA sequence (Figure 5). According to a test of the variation in the miRNAs concentration (10 pM to 100 nM were tested) of the clinical samples, the linear gradient showed R^2^ = 0.9975, indicating that the device is highly stable and that even a low dose of miRNA in can be detected in urine can (Figure 6). We further compared these results with those of similar devices. The lowest concentration detected in the 10 pM sample is the minimum value of the proposed exomiR biosensor, whereas other instruments can measure at even lower rates of about 10 aM, which is a difference of 1000 times [33]. To the best of our knowledge, unless the sensor is sufficiently stable and sensitive, usually with a lower concentration of the test substance, there is a greater interference, especially when the device detects real samples.

In the current study, we considered a single time point, either upon collection of a patient’s urine or at the time of detection. We observed that the expression level of miRNAs in urine can be altered according to time differences, which should be taken into consideration in the future.

We further compared our results with those reported by Prof. Yasui and colleagues, who aimed to analyze a whole set of miRNAs in urine [34]. They proposed a nanowire-embedded PDMS methodology, fabricated a substrate in four steps to collect urine extracellular vesicle-encapsulated miRNAs, and attempted to reveal a large number of miRNAs of different sequences in urine. The results were then derived based on microarray analysis. Although it might be an efficient device to simultaneously derive a large amount of data, big data require a considerable amount of time for further analysis, which means that large amounts of data could cause difficulty for clinicians who require immediate practical therapeutic decisions, unless the data contain certain biomarkers. With our device, our aim was to directly target cancer biomarkers; by comparing clinical urine samples among prostate cancer, benign prostatic hypertrophy, and normal subjects, we were able to obtain relevant and meaningful data.

SPCEs have been widely used for various analytical purposes. With respect to the design of SPCEs, the sensor is usually optimized by modifying its surface composition to achieve the properties of fast detection, high reproducibility, sensitivity, and accuracy of results. Depending on the detection target, different metals, enzymes, complexing agents, polymers, etc., are added to adjust the composition of the ink. The results obtained with proposed exomiR biosensor were compared with previous reports [17,27,32], and we optimized the surface of the SPCE with minor modification of the biotin-binding protein Streptavidin to NeutrAvidin, then applied it to real clinical urine samples from PCa patients, which considerably increased affinity of the probe, as well as the stability of the platform. The proposed exomiR biosensor could significantly improve the clinical screening of PCa patients, which, to date, has relied on invasive methods to collect serum to detect PSA. However, the high overdiagnosis rate and the 1.5 to 2-fold false-negative rate should always be considered.

## 5. Conclusions

With the goal of precision medicine and the enhancement of the convenience of telemedicine, a convenient device is highly necessary. The accurate detection of urinary miRNA using the proposed exomiR biosensor was verified by the consistency of serum PSA detection. Moreover, our findings through two examinations directly reflect the tumoral progression. We therefore strongly suggest this simple but highly accurate biosensor device for use in conjunction with PSA tests for the management of PCa. The proposed device may help clinicians by providing useful and immediate information, supporting quick treatment decisions.

## Figures and Tables

**Table 1:** Chi-square analysis of current values of exomiR-451 and exomiR-21 correlation and clinical pathological data of preoperative patients. (Bold *p*-values indicate significance less than 0.05).

	Current Value	exomiR-451		*p*-Value	exomiR-21		*p*-Value
Clinical Characteristics		≦*220* nA	>*220* nA		≦*220* nA	>*220* nA	
		*Case Number (n)*			*Case Number (n)*		
*Disease type*							
Adenocarcinoma		(3)	(19)	**0.005**	(4)	(18)	0.492
BPH		(2)			(1)	(1)	
Normal		(10)				(10)	
*PSA*							
≤4		(12)		**0.001**	(1)	(11)	0.504
>4		(3)	(19)		(4)	(18)	
≤10		(13)	(4)	**0.037**	(2)	(6)	0.525
>10		(2)	(15)		(3)	(14)	
*Age*							
≤63		(12)	(7)	0.455	(3)	(16)	0.301
˃63		(3)	(12)		(2)	(13)	

**Table 2:** Comparison the pre- and postoperative current values of exomiR-451 and exomiR-21 with tumor status in ten adenocarcinoma patients. (Bold *p*-values indicate significance less than 0.05).

	miRNAs	exomiR-451		exomiR-21		Comparison of	
						Current Variation	
Tumor Status		Before OP. *	After OP.	Before OP.	After OP.	exomiR-451	exomiR-21
pT stage							
T2 (5)		0.184	0.105	0.971	0.0525		
T3a (3)							
T3b (2)		0.591	0.566	**0.016**	0.907		
Lymphatic metastasis							
Yes (3)		0.218	0.158	0.15	0.504	0.789	**0.042**
No (7)							
Tumor stage							
Group 3, 4 (5)		0.985	0.591	0.811	0.352		
Group 1, 2 (5)						**0.031**	0.748

* op., operation.

## Data Availability

Not applicable.
